# Inhibition of Adenosine Pathway Alters Atrial Electrophysiology and Prevents Atrial Fibrillation

**DOI:** 10.3389/fphys.2020.00493

**Published:** 2020-06-12

**Authors:** Luca Soattin, Anniek Frederike Lubberding, Bo Hjorth Bentzen, Torsten Christ, Thomas Jespersen

**Affiliations:** ^1^Department of Biomedical Sciences, Faculty of Health and Medical Sciences, University of Copenhagen, Copenhagen, Denmark; ^2^Institute of Experimental Pharmacology and Toxicology, University Medical Center Hamburg-Eppendorf, Hamburg, Germany; ^3^DZHK (German Center for Cardiovascular Research), Partner Site Hamburg/Kiel/Lübeck, Hamburg, Germany

**Keywords:** adenosine, A_1_-R, arrhythmias, CD73, hypoxia, translational models

## Abstract

**Background:**

Adenosine leads to atrial action potential (AP) shortening through activation of adenosine 1 receptors (A_1_-R) and subsequent opening of G-protein-coupled inwardly rectifying K^+^ channels. Extracellular production of adenosine is drastically increased during stress and ischemia.

**Objective:**

The aim of this study was to address whether the pharmacological blockade of endogenous production of adenosine and of its signaling prevents atrial fibrillation (AF).

**Methods:**

The role of A_1_-R activation on atrial action potential duration, refractoriness, and AF vulnerability was investigated in rat isolated beating heart preparations (Langendorff) with an A_1_-R agonist [2-chloro-*N*^6^-cyclopentyladenosine (CCPA), 50 nM] and antagonist [1-butyl-3-(3-hydroxypropyl)-8-(3-noradamantyl)xanthine (PSB36), 40 nM]. Furthermore, to interfere with the endogenous adenosine release, the ecto-5′-nucleotidase (CD73) inhibitor was applied [5′-(α,β-methylene) diphosphate sodium salt (AMPCP), 500 μM]. Isolated *trabeculae* from human right atrial appendages (hRAAs) were used for comparison.

**Results:**

As expected, CCPA shortened AP duration at 90% of repolarization (APD_90_) and effective refractory period (ERP) in rat atria. PSB36 prolonged APD_90_ and ERP in rat atria, and CD73 inhibition with AMPCP prolonged ERP in rats, confirming that endogenously produced amount of adenosine is sufficiently high to alter atrial electrophysiology. In human atrial appendages, CCPA shortened APD_90_, while PSB36 prolonged it. Rat hearts treated with CCPA are prone to AF. In contrast, PSB36 and AMPCP prevented AF events and reduced AF duration (vehicle, 11.5 ± 2.6 s; CCPA, 40.6 ± 16.1 s; PSB36, 6.5 ± 3.7 s; AMPCP, 3.0 ± 1.4 s; *P* < 0.0001).

**Conclusion:**

A_1_-R activation by intrinsic adenosine release alters atrial electrophysiology and promotes AF. Inhibition of adenosine pathway protects atria from arrhythmic events.

## Introduction

G-protein-coupled inwardly rectifying K^+^ channels (GIRK) play a key role in the physiological regulation of the heart. GIRK channels are in part responsible for the vagus-induced negative chronotropy upon muscarinic receptor (M_2_-R) activation (Pfaffinger et al., 1985; Sato et al., 1990), and their current is therefore referred to as *I*_K,ACh_. Constitutive activation of these channels in the absence of muscarinic agonists has been implicated in human persistent AF (Dobrev et al., 2005). Apart from being activated through muscarinic receptors, recent evidence has shown that adenosine, via the A_1_-receptor (A_1_-R), also increases GIRK channel conductance (Wang et al., 2013; Li et al., 2016). In the atrium, GIRK channel activation causes resting membrane potential (RMP) hyperpolarization and shortens both action potential duration (APD) and effective refractory period (ERP). Since the shortening of APD and refractoriness represents hallmarks of pro-arrhythmicity, adenosine can evoke atrial arrhythmias in humans, as well as in animal models (Drury and Szent-Gyorgyi, 1929; Belhassen et al., 1984; Kabell et al., 1994; Bertolet et al., 1997; Strickberger et al., 1997; Tebbenjohanns et al., 1997). For this reason, adenosine can be used as a tool to detect the location of possible AF *foci* during ablation procedures (Li et al., 2016; Letsas et al., 2017).

Adenosine is a purine nucleoside produced from adenosine monophosphate (AMP) by the glycosyl phosphatidylinositol-anchored membrane ectonucleotidase CD73, catalyzing the dephosphorylation of AMP to adenosine (Colgan et al., 2006). CD73 has been associated with cardioprotection during ischemia and hypoxia (Eckle et al., 2007), as extracellular adenosine functions as a protective metabolic signal following oxygen demand (Fox et al., 1974; Haneda et al., 1989). Adenosine is released upon energy perturbations, such as ischemia or hypoxia, and its extracellular levels are subsequently fine-tuned by the activity of transporters and enzymatic cascades (Eltzschig et al., 2013). Adenosine levels are limited in time and space by a fast conversion to the metabolite inosine by adenosine deaminase, as well as adenosine reuptake and subsequent phosphorylation to AMP by the equilibrative nucleoside transporters and adenosine kinase, respectively (Moser et al., 1989; Sheth et al., 2014). During normoxia, systemic adenosine levels have been reported to be stable around ∼21 nM (Saito et al., 1999), while during cardiac injury and heart failure, adenosine levels can increase sevenfold (Funaya et al., 1997). Purinergic signaling is under control of the transcription factor hypoxia inducible factor-1α (HIF-1α) (Thompson et al., 2004; Eltzschig et al., 2013). In normoxic conditions, prolyl hydroxylases degrade HIF-1α via ubiquitine-proteasome degradation consuming oxygen, resulting in low adenosine levels (Semenza, 2011). During hypoxia, low oxygen levels reduce the activity of prolyl hydroxylases, stabilizing HIF-1α, which translocates into the cell nucleus and upregulates CD73 (Eltzschig and Carmeliet, 2011). Moreover, HIF-1α downregulates transcription of the nucleoside equilibrative transporters and adenosine deaminase (Eltzschig et al., 2013). Therefore, hypoxia increases adenosine availability, slows adenosine metabolism rate, and, thereby, increases adenosine receptor activation.

Four types of adenosine receptors are expressed in the heart (A_1_, A_2__A_, A_2__B_, and A_3_), which can also function as heterodimers in both physiological and pathological conditions (Headrick et al., 2011). A_1_-R is a G*_*i*_*-protein-coupled receptor and is predominantly expressed in atrial and nodal tissue (Pelleg et al., 1987; Belardinelli et al., 1995). In the atrioventricular (AV) node, adenosine slows the heart rate. This is partly accomplished by the activation of GIRK channels through the G*_*i*_*βγ-subunits and partly by the reduction in cyclic adenosine monophosphate (cAMP) through the G*_*i*_*α-subunit. Reduced cAMP levels decrease hyperpolarization-activated cyclic nucleotide-gated (HCN) channel activity in pacemaker cells and decrease protein kinase A activity, thereby reducing L-type calcium current (Belardinelli and Isenberg, 1983; Belardinelli et al., 1989, 1995; Pelleg et al., 1990). This process leads to a slowing of AV conduction (Belardinelli et al., 1995). For this reason, adenosine is used in clinical practice to terminate supraventricular tachycardia, such as atrioventricular nodal reentrant tachycardia (AVNRT) (DiMarco et al., 1990; Tebbenjohanns et al., 1997). However, the upregulation of A_1_-R in the failing sinoatrial (SA) node has been associated with an increase in AF in a canine heart failure model (Lou et al., 2014). AF drivers anchor to heterogeneity regions, which can also be anatomically determined (Haissaguerre et al., 2016). A large area of structural and electrical heterogeneity has been identified between the SA node complex and the surrounding right atrial tissue (Swarup et al., 2014). In addition, impairment in SA node conduction causes electrical remodeling and fibrosis, leading to micro-reentry circuits, which promote initiation and maintenance of AF (Thery et al., 1977; Kistler et al., 2004; Sanders et al., 2004).

AF is associated with heart failure and thromboembolic diseases (Rienstra et al., 2004). Moreover, AF patients’ daily life is often affected due to exertional dyspnea and reduced exercise tolerance (Hagens et al., 2004; Rienstra et al., 2004). In fact, it has been shown that oxygen uptake is significantly lowered in AF patients during exercise (Takano et al., 2019). Normoxic conditions play a fundamental role in atrial physiological function (Schuttler et al., 1983). Reduced supply of oxygen in localized atrial regions of a canine acute model showed a profound conduction slowing, which promoted reentry mechanisms (Sinno et al., 2003). In addition, more evidence highlight the role of hypoxia/ischemia in AF. In a case study of inferior infarction, AF spontaneously terminated after atrial branch reperfusion (Bunc et al., 2001). Isolated atrial tissues from AF patients showed a higher degree of fibrosis and vessel density (Gramley et al., 2010). Moreover, AF tissues express higher amount of typical hypoxic markers, such as HIF-1α and vascular endothelial growth factor (VEGF) (Gramley et al., 2010). It has also been proposed that fibrosis, upon structural remodeling in AF, might increase the diffusion distance of oxygen through the tissue and chronically trigger hypoxic and ischemic signaling (Mihm et al., 2001; Gramley et al., 2010).

In this study, we scrutinized the electrophysiological effects of intrinsic adenosine release, as well as extrinsic A_1_-R activation, in rat heart and human atrial tissue. We show that the activation of A_1_-R not only shortens atrial APD and ERP but also promotes AF susceptibility and duration. In contrast, antagonizing the activation of A_1_-R, or pharmacologically impairing the intrinsic adenosine release by inhibition of CD73, protects against arrhythmias.

## Materials and Methods

### Electrophysiological Recordings in Langendorff Preparation From Rat Hearts

**FIGURE 1:**
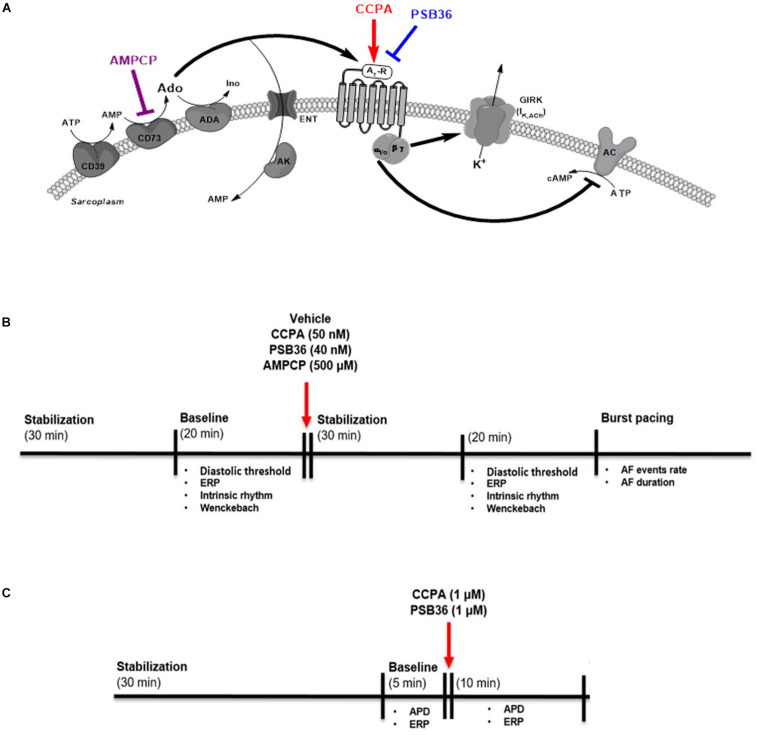
**(A)** Adenosine (Ado) pathway in cardiomyocytes. Adenosine release is regulated by the ectonucleotidases CD39 and CD73. CD39 converts ATP and ADP into AMP, while CD73 converts AMP into adenosine. Extracellular adenosine half-life is determined by adenosine deaminase (ADA) conversion to inosine (Ino) or internalization by specific equilibrative nucleoside transporters (ENT). A_1_-receptor activation increases K^+^ permeability for the G-protein-coupled inwardly rectifying K^+^ (GIRK) channel through the Gβγ and inhibits the adenylyl cyclase (AC) through G_i_α subunit. 2-Chloro-*N*^6^-cyclopentyladenosine (CCPA) is a potent A_1_-R agonist; 1-butyl-3-(3-hydroxypropyl)-8-(3-noradamantyl)xanthine (PSB36) specifically antagonizes A_1_-R signaling. 5′-(α,β-methylene) diphosphate sodium salt (AMPCP) inhibits CD73. **(B)** Experimental protocol on isolated perfused male Wistar rat hearts. Hearts were removed and placed on the Langendorff configuration. After stabilization phase and baseline, hearts were perfused with vehicle, CCPA, PSB36, or AMPCP, respectively. Each group was paced at CL of 200, 150, and 100 ms. At the end of each experiment, the hearts were exposed to high-frequency pacing events in order to induce AF. **(C)** Experimental protocol on intact contracting muscles from human right atrial appendages (hRAAs). Human atrial tissues were paced at CL of 1.0 s. After stabilization phase, baseline was recorded for 5 min. After that, hRRAs were exposed to CCPA or PSB36, respectively, for 10 min.

Animal experiments were performed according to the ethical standards of the Danish Research Animal Committee (license no. 2012/152934-00345) and in accordance with the Danish legislations. A total of 38 male Wistar rats, 300–400 g, were anesthetized with sodium pentobarbital (40 mg/kg IP). By means of a tracheostomy, the rats were ventilated (4 ml/60 strokes/min) through a ventilator (7025 Rodent ventilator, Ugo Basile, Comerio VA, Italy). To remove the hearts, a thoracotomy was performed. Through a small aortic transection close to the aortic arch, the hearts were cannulated and connected to the Langendorff retrograde perfusion system (Hugo Sachs Elektronik -Harvard Apparatus GmbH, March-Hugstetten, Germany). The hearts were perfused at a constant pressure of 80 mmHg with a 37°C, pH 7.4, Krebs–Henseleit buffer (in mmol/L) NaCl 120.0, NaHCO_3_ 25.0, KCl 4.0, MgSO_4_ 0.6, NaH_2_PO_4_ 0.6, CaCl_2_ 2.5, glucose 11.0, at constant oxygenation with a O_2_/CO_2_ mixture of 95/5%. The aforementioned devices were connected to an amplifier (Hugo Sachs Elektronik -Harvard Apparatus GmbH, Germany). Epicardial monophasic action potential (MAP) electrodes (Hugo Sachs Elektronik -Harvard Apparatus GmbH, March-Hugstetten, Germany) were placed on the left and right atria. The electrocardiogram (ECG) was obtained with three ECG electrodes (Hugo Sachs Elektronik -Harvard Apparatus GmbH, March-Hugstetten, Germany) placed in close proximity to the heart. The hearts were immersed into a temperature-controlled bath containing the above-mentioned buffer. The pacing stimuli were twice the diastolic threshold with a duration of 2 ms. A pacing electrode was placed on the atrium, and the heart was allowed to stabilize for 30 min before recording the electrophysiological parameters. After baseline measurements, each group was perfused with a drug [final concentration, 2-chloro-*N*^6^-cyclopentyladenosine (CCPA) 50 nM, 1-butyl-3-(3-hydroxypropyl)-8-(3-noradamantyl)xanthine (PSB36) 40 nM, 5′-(α, β-methylene) diphosphate sodium salt (AMPCP) 500 μM]. After stabilization, hearts were paced for 5 min with a cycle length (CL) of 200, 150, and 100 ms, respectively. At each pacing rate, diastolic threshold (DT)—applied field stimulation to elicit an action potential—was evaluated and doubled. To measure the ERP at different pacing rates, the delay (Δ_S2 – S1_) between the normal electrical stimulus (S_1_) and an additional stimulus (S_2_) was increased until an AP occurred. To measure the atrioventricular Wenckebach point—block of AV nodal conduction—the pacing rate was increased until second- and/or third-degree AV block occurred. To evaluate intrinsic rhythm, pacing was switched off for 3 min. At a CL of 100 ms, high-frequency electrical pacing (CL of 10 ms, for 500–1,000 ms, twice the DT) for inducing AF was applied up to 20 times every 3–5 s. AF episode duration was calculated from the end of electrical stimulation to the termination of the arrhythmic event—evaluated as a rapid atrial firing longer than 0.5 s. All data were acquired using the 16-channel PowerLab system (ADInstruments Ltd., Dunedin, New Zealand) (Figures 1A,B).

### Electrophysiological Recordings in Human Intact Cardiac Atrial Muscles

The investigation on human samples conforms to all principles outlined by the Declaration of Helsinki and the Medical Association of Hamburg (Germany). According to the guidelines of the ethical review committee of the Medical Association of Hamburg, there is no need for a specific approval in this case, since patient data were used anonymized. All materials from patients were taken with informed consent of the donors. Eleven intact cardiac muscles from the right atrial appendages (RAAs) were obtained from 11 patients in sinus rhythm (SR) undergoing coronary artery bypass surgery and/or valve replacement (Supplementary Material 1). Atrial APs were recorded with intracellular microelectrodes in the right atrial trabeculae (microelectrode tip resistance, 10–25 MΩ; 1-ms stimulus, 25% above DT). The bath solution contained (in mmol/L): NaCl 127.0, KCl 4.5, MgCl_2_ 1.5, CaCl_2_ 1.8, glucose 10.0, NaHCO_3_ 22.0, and NaH_2_PO_4_ 0.42, equilibrated with O_2_/CO_2_ mixture of 95/5% at 37°C, pH 7.4. All data were acquired using the PowerLab system (ADInstruments Ltd., Dunedin, New Zealand). Drugs were applied using a fast perfusion system. Tissues were allowed to stabilize for 30 min at least. Afterward, the trabeculae were electrically paced during the entire experiment at CL of 1,000 ms. Baseline was recorded for 5 min before the administration of 1 μM CCPA or 1 μM PSB36. ERP was measured at baseline and after 10 min of drug superfusion (Figure 1C).

### Compounds

Experiments were conducted using the A_1_-R agonist CCPA (Tocris Bioscience Cat. No. 1705, Abingdon, United Kingdom) dissolved in a mixture of dimethyl sulfoxide (DMSO—Sigma Aldrich Cat. No. PHR1309) and distilled water (ratio 1:100), the A_1_-R antagonist PSB36 (Tocris Bioscience Cat. No. 2019, Abingdon, United Kingdom) dissolved in a mixture of DMSO and distilled water (ratio 1:100), the CD73 inhibitor AMPCP (Tocris Bioscience Cat. No. 3633, Abingdon, United Kingdom) dissolved in distilled water. DMSO (1%) was used as vehicle (Figure 1A).

### Data Analysis

Data are presented as mean ± SEM. All Langendorff and human AP measurements data were analyzed using Labchart 7 (ADInstruments, Ltd., Dunedin, New Zealand).

Statistical analysis was performed in GraphPad Prism 7 (GraphPad Software, La Jolla, CA, United States). Two-tailed paired *t*-tests were used to compare baseline with the effect of vehicle, CCPA, PSB36, and AMPCP on intrinsic rhythm, Wenckebach point APD_90_, and ERP. All detailed statistics of isolated heart data are presented in the Supplementary Material 2. AF duration values of each group were analyzed through a relative cumulative distribution (bin center, 1.0 s) by non-parametric Kruskal–Wallis test. Following, the Dunn’s multiple comparisons posttest was used to compare vehicle with the treated groups. *P* < 0.05 were considered statistically significant (denoted by asterisks in figures). Values of *P* < 0.01 and *P* < 0.001 are denoted by ^∗∗^ and ^∗∗∗^, respectively. Schemes were drawn with ChemBioDraw Ultra 12.0 (PerkinElmer, Waltham, MA, United States).

## Results

Isolated heart studies in Langendorff configuration allow for exploration of the cardiac effects of a biochemical pathway in isolation from the rest of the body. To study the effects of adenosine signaling in the atrium, we exposed the heart to vehicle (*n* = 10), the specific A_1_-R agonist CCPA (*n* = 11), the A_1_-R antagonist PSB36 (*n* = 10), and the specific CD73 antagonist AMPCP (*n* = 7) (Figure 1).

### Effect of A_1_-R on Chronotropy

**FIGURE 2:**
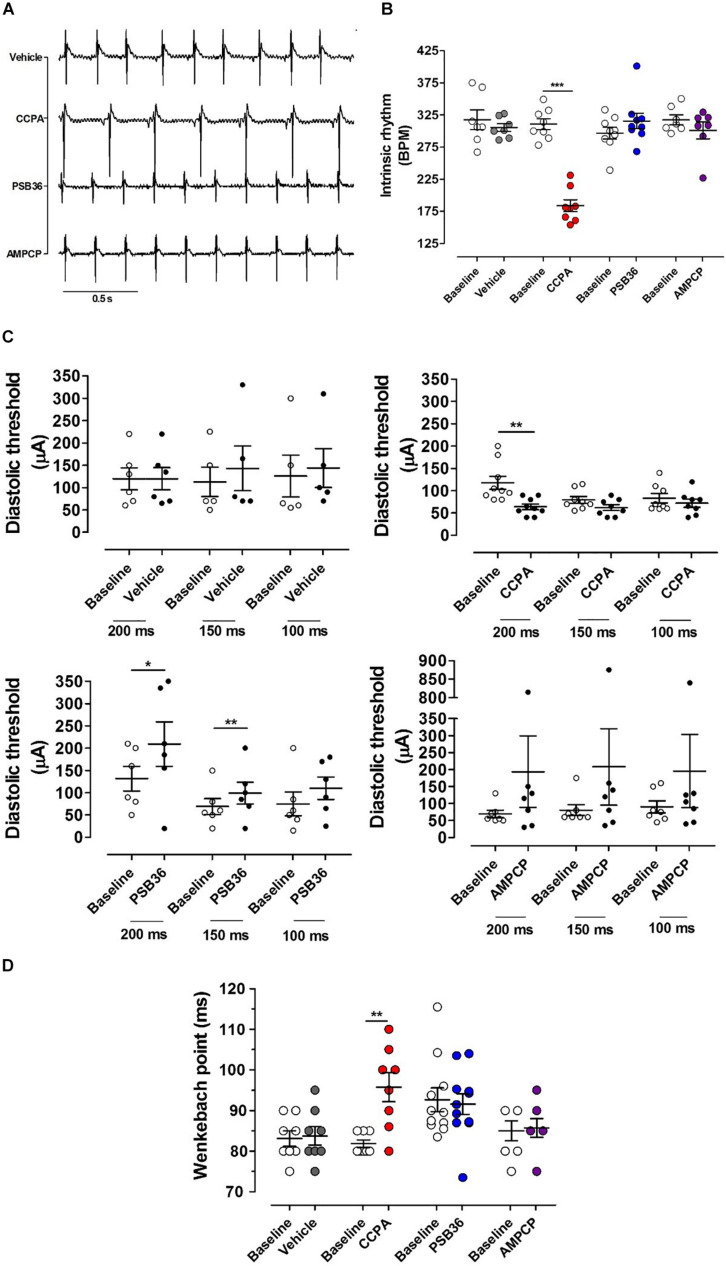
Chronotropic and dromotropic effects in rat hearts. **(A)** ECG lead II recordings under intrinsic rhythm. The R–R interval has been used to calculate heart rate. Paired *t-*tests between the baseline and the drugs were performed to evaluate significant differences. **(B)** Chronotropic effect of vehicle, 2-chloro-*N*^6^-cyclopentyladenosine (CCPA), 1-butyl-3-(3-hydroxypropyl)-8-(3-noradamantyl)xanthine (PSB36), and 5′-(α,β-methylene) diphosphate sodium salt (AMPCP) (gray, red, blue, and purple dot plots, respectively). White dot plots represent the baseline. Values are presented as mean ± SEM. **(C)** Effect of vehicle, CCPA, PSB36, and AMPCP on the diastolic threshold (DT), respectively. **(D)** Effect of vehicle, CCPA, PSB36, and AMPCP on Wenckebach’s point, respectively. Vehicle did not affect atrio-ventricular node conduction. CCPA significantly increased Wenckebach point. PSB36 slightly reduced Wenckebach’s point. AMPCP had no effect on AV node conduction. *p < 0.05, **p < 0.01, ***p < 0.001.

The intrinsic rhythm was measured by electrocardiogram (RR on ECG) (Figures 2A,B). The administration of DMSO did not affect heart rate. As expected, the A_1_-R agonist CCPA produced a profound negative chronotropic effect. In contrast, perfusion with the specific A_1_-R antagonist PSB36 produced a non-significant positive chronotropic effect (*P* = 0.056). Increased concentration of both CCPA (>50 nM) and PSB36 (>80 nM) produced runs of tachycardia as well as other arrhythmic activities (data not shown). No significant difference in beating rate was detected when the 5′-ecto-nucleotidase CD73 was inhibited with AMPCP (Figures 2A,B).

### Diastolic Threshold

The diastolic threshold (DT) is a measure of excitability of the atrial tissue (Figure 2C). While DT did not change in the vehicle group, the perfusion with CCPA increased membrane excitability, showing a significant DT reduction when the heart was paced at cycle length (CL) of 200 ms and a tendency toward reduction in DT at CL of 150 and 100 ms. By contrast, PSB36 increased DT at CLs of 200 and 150 ms. AMPCP-treated atria showed a tendency toward increased DT; however, this did not reach significance.

### Wenckebach Point

To detect whether targeting of the purinergic system affects the electrical conduction between atria and ventricles, we increased the pacing rate until the atrioventricular Wenckebach point was reached. Hearts treated with vehicle did not show any difference from baseline. Not surprisingly, the activation of A_1_-R with CCPA prolonged the Wenckebach point. PSB36, antagonizing A_1_-R, showed a slight tendency in reducing the Wenckebach point, while no effect was observed on the atrioventricular conduction when CD73 was inhibited with AMPCP (Figure 2D).

### Action Potential Duration

**FIGURE 3:**
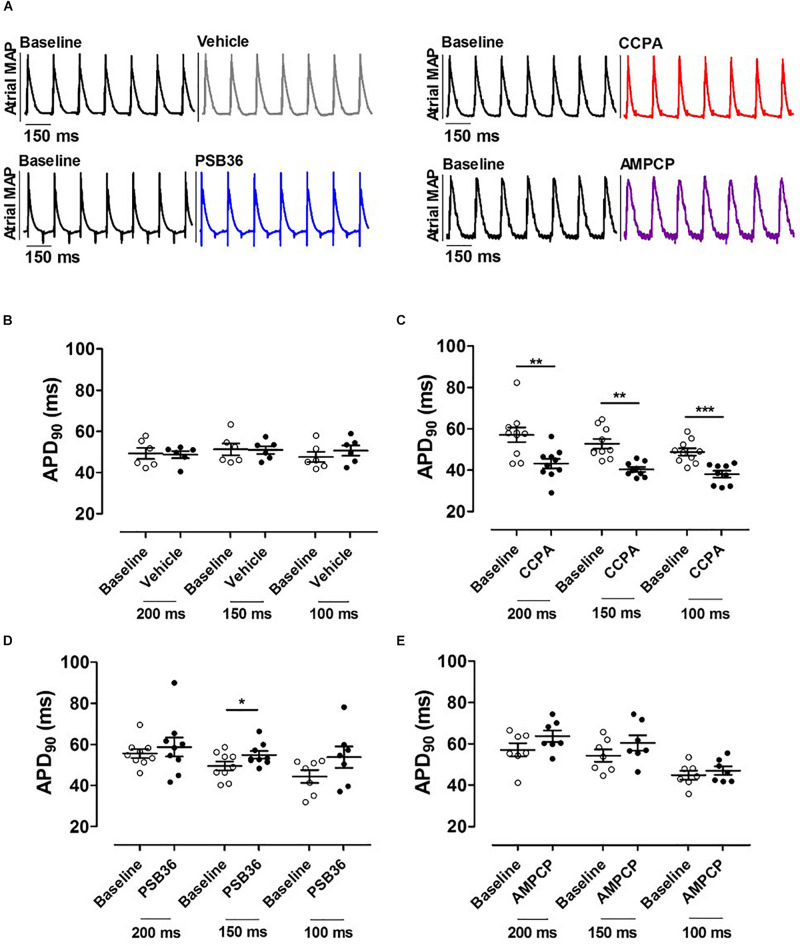
**(A)** Atrial monophasic action potentials (MAPs) paced at cycle length (CL) of 150 ms before and after the administration of vehicle, 2-chloro-*N*^6^-cyclopentyladenosine (CCPA), 1-butyl-3-(3-hydroxypropyl)-8-(3-noradamantyl)xanthine (PSB36), or 5′-(α,β-methylene) diphosphate sodium salt (AMPCP); black MAPs refer to the baseline, while gray, red, blue, and purple MAPs represent hearts perfused with vehicle, CCPA, PSB36, and AMPCP, respectively. **(B)** Effect of vehicle on APD_90_ at CL of 200, 150, and 100 ms. **(C)** CCPA shortened APD_90_ at CL of 200, 150, and 100 ms, respectively. **(D)** PSB36 showed a prolongation of APD_90_ at CL of 200, 150, and 100 ms. **(E)** Effect of AMPCP on APD_90_ at CL of 200, 150, and 100 ms. *p ¡ 0.05, **p < 0.01, ***p < 0.001.

To investigate the effect on APD at 90% of repolarization (APD_90_) in the atria, the hearts were paced at CLs of 200, 150, and 100 ms for 5 min (Figure 3A). As expected, APD_90_ moderately decreased as pacing decreased (Supplementary Material 3). Atrial APD was not altered by the administration of the vehicle (Figure 3B). CCPA provoked a profound APD_90_ reduction at CL of 200, 150, and 100 ms (Figure 3C and Supplementary Material 4). CCPA showed a maximum APD_90_ shortening of ∼51% in dose–response relationship tests (Supplementary Material 5).

On the other hand, PSB36 showed a tendency to prolong the atrial APD_90_ in a rate-dependent manner (Supplementary Material 4). The prolongation was significant at CL of 150 ms (Figure 3D). The inhibition of CD73 showed a tendency in APD_90_ increase (Figure 3E).

### Effective Refractory Period

**FIGURE 4:**
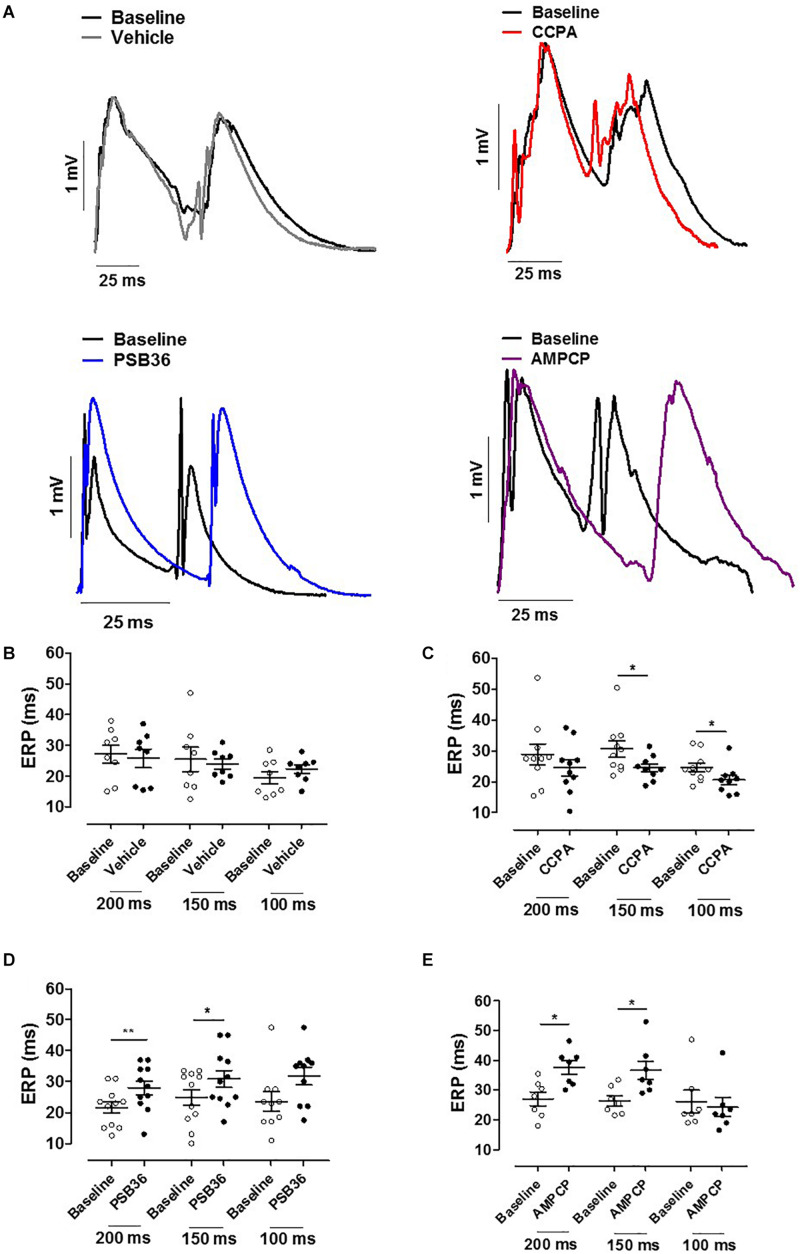
**(A)** Effective refractory period (ERP) monophasic action potentials (MAPs) of baseline and the relative treated hearts are shown superimposed. **(B)** Effect of vehicle on ERP at cycle length (CL) 0.20, 0.15, and 0.10 s. **(C)** 2-Chloro-*N*^6^-cyclopentyladenosine (CCPA) shortened refractoriness at CL of 200 ms, significantly at CL of 150 and 100 ms. **(D)** 1-Butyl-3-(3-hydroxypropyl)-8-(3-noradamantyl)xanthine (PSB36) prolonged ERP at CL of 200 and 150 ms. **(E)** 5′-(α,β-Methylene) diphosphate sodium salt (AMPCP) prolonged ERP at CL of 200 and 150 ms, but not at 100 ms. *p < 0.05, **p < 0.01.

Hearts treated with vehicle did not change ERP over time, while CCPA significantly shortened ERP at CLs of 150 and 100 ms (Figures 4A–C). A_1_-R inhibition by PSB36 showed the opposite effect, prolonging ERP significantly in a rate-dependent manner at CLs of 200 and 150 ms, and with a tendency at CL of 100 ms (Figure 4D). AMPCP induced a significant prolongation of refractoriness at CLs of 200 and 150 ms, but not at CL of 100 ms (Figure 4E).

### Induction of Atrial Fibrillation

**FIGURE 5:**
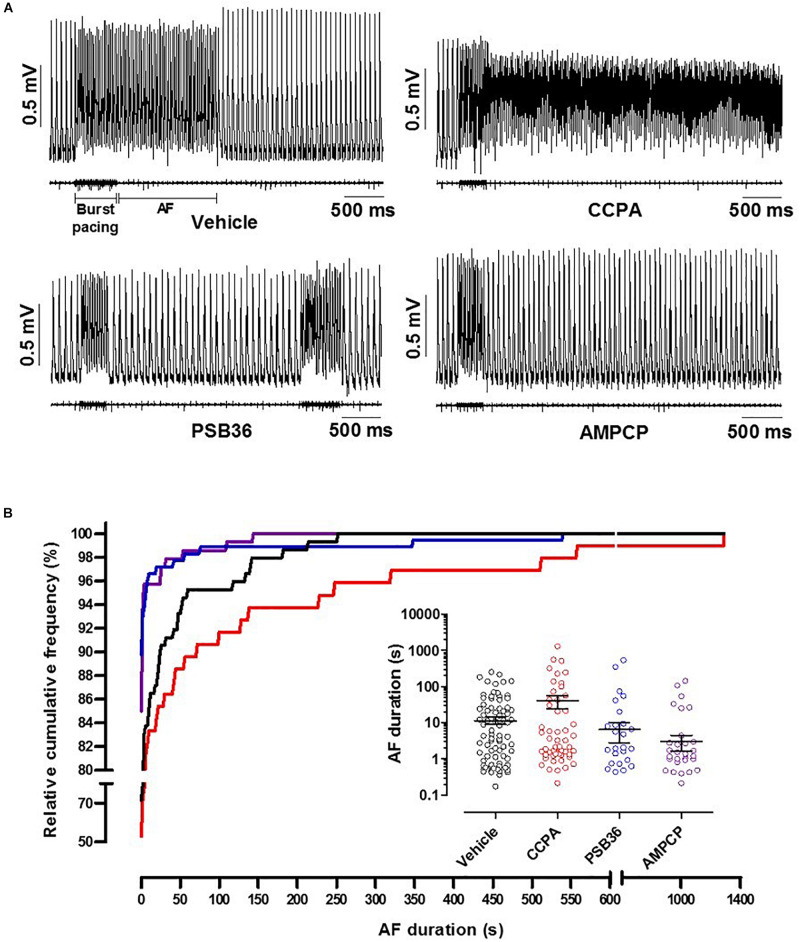
Induction of atrial fibrillation (AF) through high-frequency electrical pacing. **(A)** Atrial monophasic action potentials (MAPs) and pacing traces are shown. Vehicle produced short AF events; by contrasts, 2-chloro-*N*^6^-cyclopentyladenosine (CCPA) made the heart prone to sustained AF events. 1-Butyl-3-(3-hydroxypropyl)-8-(3-noradamantyl)xanthine (PSB36) and 5′-(α,β-methylene) diphosphate sodium salt (AMPCP) reduced atrial sensitivity to high frequency electrical pacing (Supplementary Material 6; *p* < 0.0001). **(B)** Relative cumulative distribution (in%) of AF duration of each group and dot plot of AF duration events on a logarithmic scale, which shows the range of AF duration among groups when events are different from 0 s. Vehicle, CCPA, PSB36, and AMPCP are depicted in black, red, blue, and purple, respectively.

To induce AF, the hearts were exposed to fast electrical pacing (Figure 5A). AF duration for hearts treated with vehicle (11.5 ± 2.6 s, *n* = 9) were compared to CCPA (40.6 ± 16.1 s, *n* = 10), PSB36 (6.5 ± 3.7 s, *n* = 10), and AMPCP (3.0 ± 1.4 s, *n* = 7). PSB36 and AMPCP significantly shortened the duration of AF events compared to vehicle, while CCPA increased the duration of AF. The relative cumulative stratification (in %) of AF event duration frequencies for each group showed that ∼90% of PSB36 and 85% of AMPCP-treated hearts had AF events lasting <2 s, in contrast to ∼72% of vehicle and only ∼53% of CCPA hearts (*P* < 0.0001 within the groups) (Figure 5B and Supplementary Material 6). In addition, spontaneous AF (sAF) events occurred more frequently in CCPA-treated hearts than in the PSB36 and AMPCP groups (Supplementary Material 7). Moreover, when atria were not paced, the duration of sAF events in PSB36 and AMPCP groups was drastically reduced and significantly lower compared to CCPA-treated hearts (*P* < 0.05) (Supplementary Material 8).

### Action Potential Duration in Human Right Atrial Appendage Trabeculae

**FIGURE 6:**
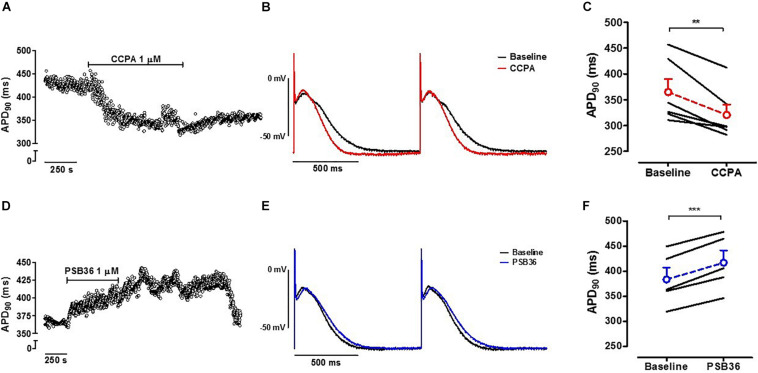
**(A)** A_1_-R activation in human right atrial appendages (hRAAs). APD_90_ measurements are shown over time when an isolated contracting atrial muscle was superfused with 1 μM 2-chloro-*N*^6^-cyclopentyladenosine (CCPA). **(B)** Baseline and CCPA-treated hRAA action potentials (APs) were superimposed to show the APD shortening due to A_1_-R activation. hRAA was paced at cycle length (CL) of 1.0 s. **(C)** CCPA shortened APD_90_ in hRAAs. **(D)** A_1_-R blockade in hRAAs. APD_90_ measurements are shown over time when an isolated contracting atrial muscle was superfused with 1 μM 1-butyl-3-(3-hydroxypropyl)-8-(3-noradamantyl)xanthine (PSB36). hRAA was paced at CL of 1,000 ms. **(E)** Baseline and PSB36-treated hRAA APs were superimposed to show the APD prolongation due to A_1_-R blockade. **(F)** PSB36 prolonged APD_90_ in hRAAs. Resting membrane potential (RMP) in representative panels **(B,E)** was normalized to show change in AP duration. **p < 0.01, ***p < 0.001.

Action potential measurements were conducted on intact contracting muscles isolated from the right atrium appendage of patients in SR. After stabilization, 1 μM CCPA or PSB36 were superfused for 10 min (Figure 1C). CCPA produced a relative APD_90_ shortening of ∼12% (Supplementary Material 9) when paced at CL of 1,000 ms (baseline 364.9 ± 25.3 ms vs. CCPA 320.5 ± 20.2 ms, *P* < 0.01) (Figures 6A–C). A_1_-R inhibition by PSB36 showed a relative APD_90_ prolongation of ∼9% (baseline 383.6 ± 23.5 ms vs. PSB36 416.9 ± 24.5 ms, *P* < 0.001; Figures 6D–F). ERP recordings on isolated human trabeculae are technically demanding, as extra stimuli often result in loss of seal. Collected data and statistics are shown in Supplementary Material 10.

## Discussion

Adenosine plays a key role in cardiac electrophysiology, which becomes especially prominent during hypoxia or ischemia. In the heart, adenosine activity is mediated by the aforementioned four types of adenosine receptors (Headrick et al., 2011). Although A_2__A_-R also has been associated with AF (Hove-Madsen et al., 2006; Llach et al., 2011; Molina et al., 2016), the present manuscript focuses on A_1_-R as the most likely candidate involved in the relation between adenosine and AF (Belardinelli and Isenberg, 1983; Visentin et al., 1990; Kabell et al., 1994; Atienza et al., 2006; Ip et al., 2013; Li et al., 2016). In fact, A_1_-R has a higher expression in the atrium and nodal tissue compared to the other adenosine receptors (Headrick et al., 2011; Lou et al., 2014). Generally, A_1_-R and A_2__A_-R are activated at nanomolar concentrations of adenosine and therefore show the highest affinity for adenosine compared to A_2__B_-R and A_3_-R (low-affinity receptors), which exhibit their activity during hypoxic stress, when adenosine levels elevate drastically (Fredholm et al., 2001; Latini and Pedata, 2001; Pedata et al., 2001). In addition, it has also been shown that A_1_-R and A_3_-R stimulation would neutralize A_2__A_-R signaling in the heart (Talukder et al., 2002; Headrick et al., 2011).

We used the specific A_1_-R agonist (CCPA) and antagonist (PSB36) that have potent and strong selectivity for A_1_-R and have very poor or no selectivity for the other adenosine receptors (Muller and Jacobson, 2011). Off-target effects through the other adenosine receptors are therefore unlikely in the present study.

Activation of A_1_-R increases potassium conductance through GIRK channels. These channels also play a pivotal role in the nodal tissue. Our experiments confirm that A_1_-R activation also activates GIRK channels in the nodes, as A_1_-R stimulation with CCPA showed a clear negative chronotropic effect and an increase in the Wenckebach point in rat isolated hearts. As previously shown, when A_1_-R is genetically deleted, the drop in heart rate normally induced by adenosine infusion is abolished (Koeppen et al., 2009). In our work, the inhibition of A_1_-R by PSB36 did not produce neither significant nor biologically relevant elevation in intrinsic rate (*P* = 0.056) of isolated perfused hearts, indicating a minor, or absent, effect of endogenous produced adenosine on the sinus node function. In addition, the Wenckebach point was not altered following PSB36 infusion, indicating that the AV node is also not affected by endogenous adenosine.

In the atria, we found that the activation of A_1_-R with CCPA profoundly shortened APD_90_ in both explanted rat hearts and human *trabeculae*. ERP was also shortened by CCPA in rat hearts. Several studies have shown that the prolonged stimulation of A_1_-R triggers desensitization of the receptors due to inactivation and internalization mechanisms (Saura et al., 1998; Klaasse et al., 2008). However, in our experiments, we did not observe effects indicating A_1_-R desensitization (Supplementary Material 11). Although it is not possible to exclude the role of other potassium channels, the observed effects at the beginning of the action potential might reflect changes in early *I*_K,ACh_ (Tang et al., 2015). For instance, activation or inhibition of A_1_-R could also trigger fluctuations of cAMP levels, which might affect L-type calcium channels (Headrick et al., 2011). However, minor changes were detected on the action potentials in our experiments. Moreover, it has been shown that the adenosine-induced decrease in *I*_Ca_ currents, via cAMP, would have a limited or no effect on the shortening of the action potential (Visentin et al., 1990).

In our work, the relative APD_90_ shortening appeared more profound in rats than in humans. Recently, Li et al. (2016) showed that in humans A_1_-R and GIRK4 protein expression follows a gradient with a maximum level in the superior lateral region of the right atrium. While we only had access to the right atrial appendage from SR patients, exposure to CCPA revealed a significant AP shortening. Interestingly, PSB36 prolonged the APD_90_ in both rat and human atria. In addition, the direct antagonism of A_1_-R prolonged ERP in isolated rat hearts. This suggests that adenosine is intrinsically released and contributes to fine-tuning of the repolarization phase of the atrial action potential. Several groups have already described intrinsic and endogenous release of adenosine (Mubagwa et al., 1996; Headrick et al., 2003; Jammes et al., 2015). To confirm the electrophysiological effect of this phenomenon, we targeted the upstream adenosine releasing mechanism with the selective CD73 inhibitor (AMPCP). AMPCP had a tendency to prolong APD_90_ and significantly prolonged ERP, suggesting that adenosine is intrinsically released under the chosen experimental conditions.

Atrial fibrillation events were induced with an average duration of ∼12 s in rat hearts treated with vehicle. The A_1_-R agonist (CCPA) made the hearts more prone to AF, increasing the duration of AF to ∼41 s. In addition, spontaneous AF events occurred more frequently in CCPA-treated hearts rather than in the PSB36 and AMPCP groups. Both the direct antagonism of the A_1_-R with PSB36 and the inhibition of the CD73 with AMPCP profoundly reduced the ability to induce sustained AF events.

Interestingly, the activation of A_1_-R with CCPA significantly lowered the DT. This could be explained by an increased GIRK channel activity, hyperpolarizing the resting membrane potential (Supplementary Material 12A), thereby increasing the fraction of available sodium channels. Such an increased sodium current, combined with shorter refractory periods, would be expected to increase vulnerability and sustainability of AF. In contrast, antagonizing A_1_-R with PSB36 produced a profound reduction in atrial excitability, represented by an increase in DT. This could suggest an increased protection from ectopic firing or focal partial depolarization. Thus, antagonizing A_1_-R with PSB36 produces an antiarrhythmic state by prolonging APD and ERP, potentially supported by a depolarization of RMP (Supplementary Material 12B), thereby reducing the availability of sodium channels and thereby susceptibility to triggered activity (Wang et al., 2013).

During hypoxic and ischemic injury, adenosine reduces energy consumption and adapts the tissue to hypoxic conditions (Eltzschig et al., 2013). Based on this, it can be speculated that the shortening of APD in the atrium, evoked by A_1_-R activation resembles a protective mechanism to avoid calcium overload during cardiac metabolic stress (Cole et al., 1991; Findlay, 1994). However, even though adenosine protects from ischemic and hypoxic heart injuries in the ventricles, hypoxic conditions in the atria may constitute a substrate for AF through A_1_-R activation. Hypoxia and ischemia in the heart are cause of excessive energy consumption. To make matters worse, the high frequency of atrial excitation and contraction in AF increases oxygen consumption while limiting oxygen supply to the tissue (Harada et al., 2017). In this setting, atrial A_1_-R activation, especially in regions with high density of GIRK channels, may constitute an important promoting and sustaining factor for AF. Recent clinical trials with *I*_K,ACh_ blockers did not show a reduction in paroxysmal AF (Podd et al., 2016). However, it has been shown that obstructive or central sleep apnea causes AF and atrial remodeling. The reiterated oxygen drop due to sleep apnea may recruit the purinergic pathway leading to A_1_-R stimulation in the atrium as AF substrate (Caples and Somers, 2009; Zhang et al., 2015). Thus, blocking endogenous adenosine production or inhibiting A_1_-R activation could be a potential treatment modality of AF. Pilot studies in rat Langendorff hearts indicated that, during AF, the acute A_1_-R blockade might restore the sinus rhythm (Supplementary Material 13). However, due to its relatively ubiquitous expression, targeting the purinergic signaling may have undesirable consequences on multiple related pathways and organs (Jacobson and Gao, 2006). Therefore, thorough electrophysiological investigations of adenosine block and A_1_-R inhibition are needed in intact AF animal models. In addition, further studies on energy consumption and calcium handling upon A_1_-R stimulation could better clarify the substantial role of the purinergic system in atrial electrophysiology.

Targeting CD73 for AF treatment appears to be promising. CD73 inhibition prolonged refractoriness and protected rat atria from sustained arrhythmogenic events. CD73 has been also identified as a target for pharmacotherapy strategies of breast cancer (Stagg et al., 2010), and it plays critical role during injury protection in the renal proximal tubule as well as in immune processes, such as inflammation (Antonioli et al., 2013; Sung et al., 2017). Furthermore, adenosine modulates vascular homeostasis, also in the heart (Koszalka et al., 2004; Headrick et al., 2011). Hence, non-beneficial side effects might limit the efficacy of CD73 as a target for AF treatment.

## Study Limitations

The use of rodents as translational model for AF carries study limitations regarding differences with human physiology. To repolarize the action potential, unlike humans, rodents rely on *I*_K,to_ currents, which accelerate the repolarization rate and shorten the plateau phase (Roden, 1998; Guo et al., 1999). However, sufficient parallels in pathophysiology of cardiac diseases have been found between rodents and humans, so that rodents can be considered a valid model for cardiac electrophysiology and AF (Nishida et al., 2010; Diness et al., 2011; Farraj et al., 2011; Skibsbye et al., 2011, 2015).

Although the number of patients represents an important limitation to this study in terms of statistical and clinical significance, human trabeculae in SR were a useful investigation tool to validate data obtained in rat isolated perfused hearts showing an important translational level of confidence between rodents and human physiology.

The use of Krebs–Henseleit and Tyrode buffers for Langendorff and Steiert organ bath experiments, respectively, may have generated a slight hypoxic environment over time. This may have contributed to the intrinsic release of adenosine. However, hypoxic conditions cannot be assessed in the systems used in this study. During perfusion and superfusion experiments of vehicle, CCPA, or PSB36, the effect of the intrinsic adenosine release on A_1_-R was not measured. However, adenosine’s half-life is extremely short, and the dissociation equilibrium constants between adenosine, CCPA, and PSB36 for A_1_-R (700, 0.4, and 0.7 nM, respectively) (Lohse et al., 1988; Bilkei-Gorzo et al., 2008; de Lera Ruiz et al., 2014) would favor the binding of CCPA or PSB36 to A_1_-R rather than the natural occurring purine nucleoside. The choice of drugs concentration was based on previous laboratory data (not shown) and recent literature.

## Conclusion

In this study, we highlighted the role of adenosine signaling in atrial electrophysiology and AF susceptibility. Reducing A_1_-R activation, either by direct inhibition or by interfering with endogenous adenosine release, produced an antiarrhythmic state of the atria. These data reveal CD73 as a potential pharmacological target in the setting of AF.

## Data Availability Statement

The datasets analyzed in this article are not publicly available. Requests to access the datasets should be directed to luca.soattin1986@gmail.com; thojes@sund.ku.dk.

## Ethics Statement

The studies involving human participants were reviewed and approved by Declaration of Helsinki and the Medical Association of Hamburg (Germany). The patients/participants provided their written informed consent to participate in this study. The animal study was reviewed and approved by Danish Research Animal Committee (license n. 2012/152934-00345).

## Author Contributions

All authors listed have made a substantial, direct and intellectual contribution to the work, and approved it for publication.

## Conflict of Interest

The authors declare that the research was conducted in the absence of any commercial or financial relationships that could be construed as a potential conflict of interest.
