# Supplementary material for: Inhibition of Adenosine Pathway Alters Atrial Electrophysiology and Prevents Atrial Fibrillation
Source: Front Physiol. 2020 Jun 12;11:493. doi: 10.3389/fphys.2020.00493 (PMC7304385; doi:10.3389/fphys.2020.00493)
Supplement: Supplementary file 1 [file Data_Sheet_1.PDF]

Supplementary Material

Human samples	Sex	Age	Disease	Surgical procedure
RAA	m	75	Aortic valve stenosis III˚<br>Coronary disease	Aortic valve replacement<br>2 x coronary artery bypass
RAA	m	55	Coronary disease	3 x coronary artery bypass
RAA	f	74	Coronary disease	1 x coronary artery bypass
RAA	m	64	Coronary disease	1 x coronary artery bypass
RAA	m	58	Coronary disease	3 x coronary artery bypass
RAA	f	62	Coronary disease	3 x coronary artery bypass
RAA	m	43	Valve disease<br>Aortic disease	Valve-sparing root re-implantation<br>Replacement of ascending aorta
RAA	m	60	Valve disease	1 x coronary artery bypass
RAA	-	-	Left ventricle ejection fraction 10%<br>Mitral regurgitation (II-III˚)<br>Borderline myocarditis	Left Ventricle Assist Device-implantation
RAA	m	58	Mitral regurgitation (III˚)<br>Coronary disease	Mitral valve reconstruction<br>1 x coronary artery bypass
RAA	m	69	Coronary disease	2 x coronary artery bypass

Supplementary Material 1. Patients' clinical data.

Chronotropic effect of vehicle, CCPA, PSB36 and AMPCP

Vehicle (baseline 318 ± 15 BPM vs. vehicle 305 ± 6 BPM, P>0.05, n=7).
CCPA (baseline 311 ± 8 BPM vs. CCPA 184 ± 9 BPM, P<0.0001, n=8).
PSB36 (baseline 297 ± 9 BPM vs. PSB36 316 ± 12 BPM, P=0.056, n=9).
AMPCP (baseline 317 ± 7 BPM vs. AMPCP 301 ± 13 BPM, P>0.05, n=7)

Effect of vehicle, CCPA, PSB36 and AMPCP on diastolic threshold (DT)

	CL: 200 ms	CL: 150 ms	CL: 100 ms
**Vehicle**	baseline 120 ± 25 µA vs. vehicle 120 ± 25 µA, P>0.05, n=6	baseline 113 ± 33 µA vs. vehicle 143 ± 50 µA, P>0.05, n=5	baseline 126 ± 47 µA vs. vehicle 144 ± 44 µA, P>0.05, n=5
**CCPA**	baseline 118 ± 15 µA vs CCPA 64 ± 6 µA; P<0.01, n=9	baseline 79 ± 8 µA vs. CCPA 62 ± 7 µA, P>0.05, n=8	baseline 83 ± 11 µA vs. CCPA 72 ± 9 µA, P>0.05, n=8
**PSB36**	baseline 132 ± 28 µA vs. PSB36 209 ± 50 µA; P<0.05, n=6	baseline 69 ± 18 µA vs. PSB36 99 ± 25 µA; P<0.01, n=6	baseline 75 ± 27 µA vs. PSB36 110 ± 25 µA, P>0.05, n=6
**AMPCP**	baseline 69 ± 11 µA vs. AMPCP 194 ± 105 µA; P>0.05, n=7	baseline 81 ± 16 µA vs. AMPCP 208 ± 17 µA; P>0.05, n=7	baseline 90 ± 17 µA vs. AMPCP 196 ± 108 µA; P>0.05, n=7

Effect of vehicle, CCPA, PSB36 and AMPCP on Wenckebach point

Vehicle (baseline 83.0 ± 2.0 ms vs. vehicle 84.0 ± 2.0 ms, P>0.05, n=8).
CCPA (baseline, 82.0 ± 1.0 ms vs. CCPA, 96.0 ± 4.0 ms; P<0.001, n=8).
PSB36 (baseline, 93.0 ± 3.0 ms vs. PSB36, 92.0 ± 3.0 s, P>0.05, n=11).
AMPCP (baseline, 85.0 ± 2.0 ms vs. AMPCP, 86.0 ± 2.0 ms, P>0.05, n=7).

Effect of vehicle, CCPA, PSB36 and AMPCP on APD_{90}

	CL: 200 ms	CL: 150 ms	CL: 100 ms
**Vehicle**	baseline 49.3 ± 2.7 ms vs. vehicle 48.7 ± 1.8 ms, P>0.05, n=6	baseline 51.3 ± 2.9 ms vs. vehicle 51.0 ± 1.9 ms, P>0.05, n=6	baseline 47.6 ± 2.5 ms vs. vehicle 50.7 ± 2.5 ms, P>0.05, n=6
**CCPA**	baseline 57.1 ± 3.6 ms vs. CCPA 43.1 ± 2.3 ms, P<0.01, n=10	baseline 52.8 ± 2.3 ms vs. CCPA 40.3 ± 1.2 ms, P<0.01, n=9	baseline 48.9 ± 1.7 ms vs. CCPA 38.0 ± 1.6 ms, P<0.001, n=9
**PSB36**	baseline, 55.6 ± 2.2 ms vs. PSB36, 58.8 ± 4.7 ms, P>0.05, n=9	baseline 49.5 ± 2.2 ms vs. PSB36 54.9 ± 1.8 ms, P<0.05, n=9	baseline 44.4 ± 3.2 ms vs. PSB36 53.9 ± 5.2 ms, P>0.05, n=7
**AMPCP**	baseline 57.2 ± 3.3 ms vs. AMPCP 63.7 ± 2.8 ms, P>0.05, n=7	baseline 54.4 ± 3.0 ms vs. AMPCP 60.5 ± 3.7 ms, P>0.05, n=7	baseline 44.9 ± 2.2 ms vs. AMPCP 47.1 ± 2.1 ms, P>0.05, n=7

Effect of vehicle, CCPA, PSB36 and AMPCP on ERP

	CL: 200 ms	CL: 150 ms	CL: 100 ms
**Vehicle**	baseline 27.2 ± 3.0 ms vs. vehicle 25.8 ± 3.0 ms, P>0.05, n=8	baseline 25.5 ms ± 4.0 ms vs. vehicle 23.9 ± 1.6 ms, P>0.05, n=8	baseline 19.4 ± 2.1 ms vs. vehicle 22.3 ± 1.4 ms, P>0.05, n=8
**CCPA**	baseline 28.9 ± 3.4 ms vs. CCPA 24.5 ± 2.6 ms, P>0.05, n=10	baseline 30.7 ± 2.6 ms vs. CCPA 24.5 ± 1.3 ms; P<0.05, n=9	baseline 24.7 ± 1.4 ms vs. CCPA 20.7 ± 1.5 ms, P<0.05, n=9
**PSB36**	baseline 21.6 ± 1.9 ms vs. PSB36 28.1 ± 2.4 ms, P<0.01, n=11	baseline 24.9 ± 2.6 ms vs. PSB36 30.9 ± 2.8 ms; P<0.05, n=11	baseline 23.6 ± 3.1 ms vs. PSB36 31.8 ± 2.9 ms, P=0.06, n=10
**AMPCP**	baseline 27.0 ± 2.4 ms vs. AMPCP 37.6 ± 2.3 ms; P<0.05, n=7	baseline 26.4 ± 1.8 ms vs. AMPCP 36.6 ± 3.2 ms; P<0.05, n=7	baseline 26.2 ± 3.8 ms vs. AMPCP 24.3 ± 3.2 ms, P>0.05, n=7

Supplementary Material 2. Data and statistics from explanted perfused rat hearts.

a) b)

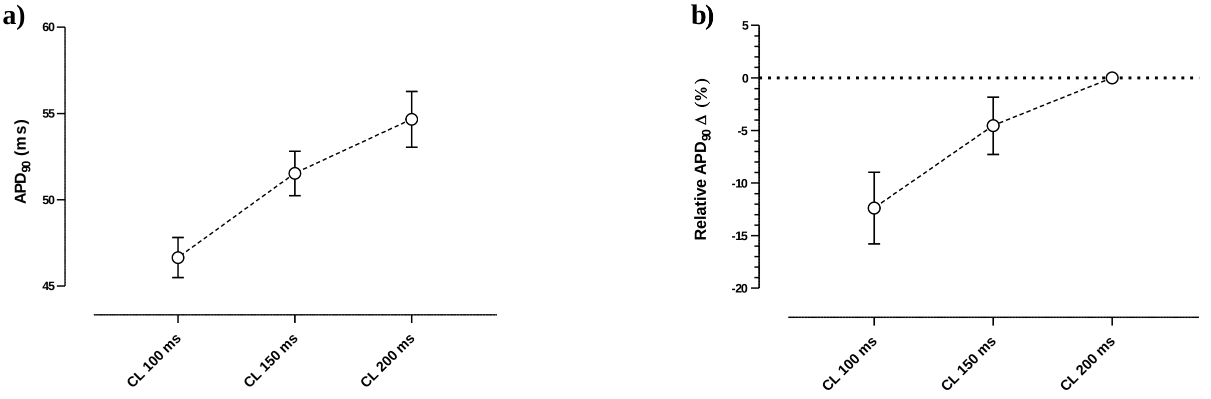

Supplementary Material 3. Atrial relative APD_{90} change. **a)** Atrial restitution curve of the Baselines APD_{90} at CL of 200, 150 and 100 ms. **b)** Restitution curve of the Baselines relative APD_{90} variation at CL of 150 (4.6 ± 2.7%, n=32) and 100 ms (12.4 ± 3.4%, n=30) compared with CL 200 ms in rat atria.

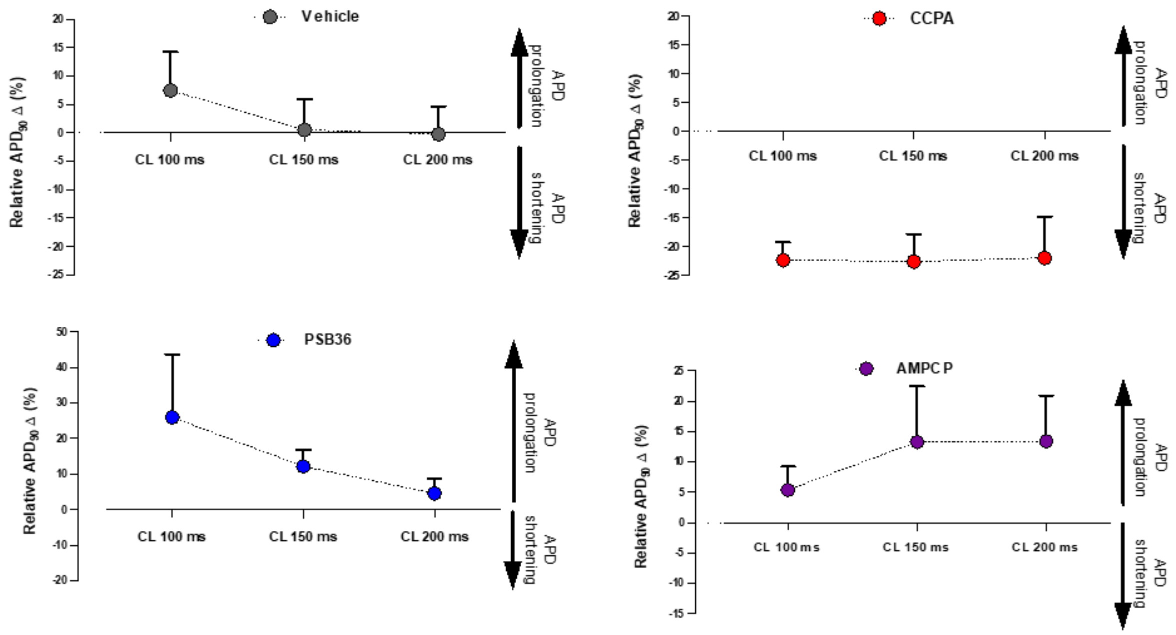

Supplementary Material 4. Relative APD90 variation of vehicle, CCPA, PSB36 and AMPCP compared with the Baseline depending on the pacing frequency (at 200, 150 and 100 ms CL) in rat atria.

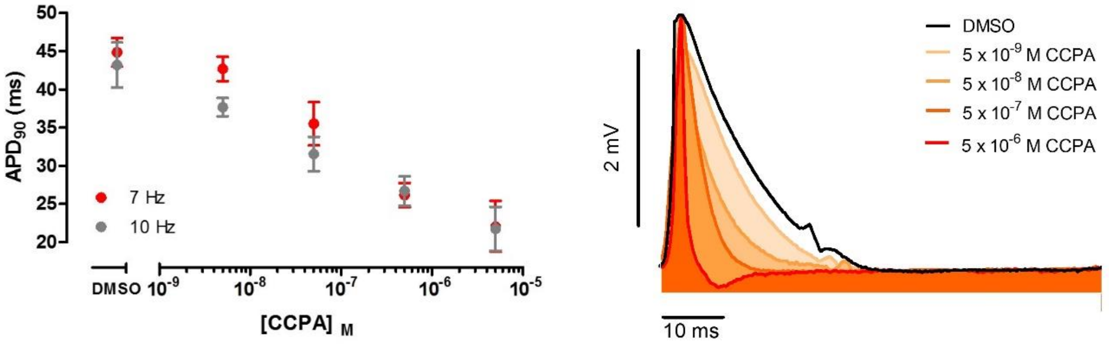

Supplementary Material 5. CCPA dose-response curve on rat atrial APD90 (n=6) at CL of 145 ms (7 Hz) and 100 ms (10 Hz). As pictorial example, MAPs are superimposed and area under the curve is depicted with increasing shade of orange, as CCPA concentration increases.

AF duration (s)	**Relative cumulative frequency (%)**			
	**Vehicle**	**CCPA**	**PSB36**	**AMPCP**
**0 - 2**	71,6	53,1	89,8	85,0
**< 50**	93,2	88,5	97,7	97,9
**< 100**	95,3	91,7	98,9	98,6
**< 200**	98,6	93,8	98,9	100,0
**< 400**	100,0	99,0	100,0	100,0
**< 1000**	100,0	99,0	100,0	100,0
**< 1400**	100,0	100,0	100,0	100,0

Supplementary Material 6. Relative cumulative distribution (%) of AF duration events at different duration times (bin center: 1.0 s). AF duration events in the group of hearts treated with vehicle (11.5 ± 2.6 s, n=9) were compared with the relative distributions of CCPA (40.6 ± 16.1 s, n=10), PSB36 (6.5 ± 3.7 s, n=10) and AMPCP (3.0 ± 1.4 s, n=7). CCPA showed the highest mean rank (m.r.) difference of 1016 (vehicle 1558 m.r. vs. CCPA 542.4 m.r., p<0.0001), while PSB36 (vehicle 1558 m.r. vs. PSB36 1370 m.r., p<0.0001) and AMPCP (vehicle 1558 m.r. vs. AMPCP 1700 m.r., p<0.001) showed a difference of 188.5 and -142.1, respectively. Therefore, PSB36 and AMPCP significantly lower the duration of AF events compared to the vehicle, while CCPA increases the duration of AF.

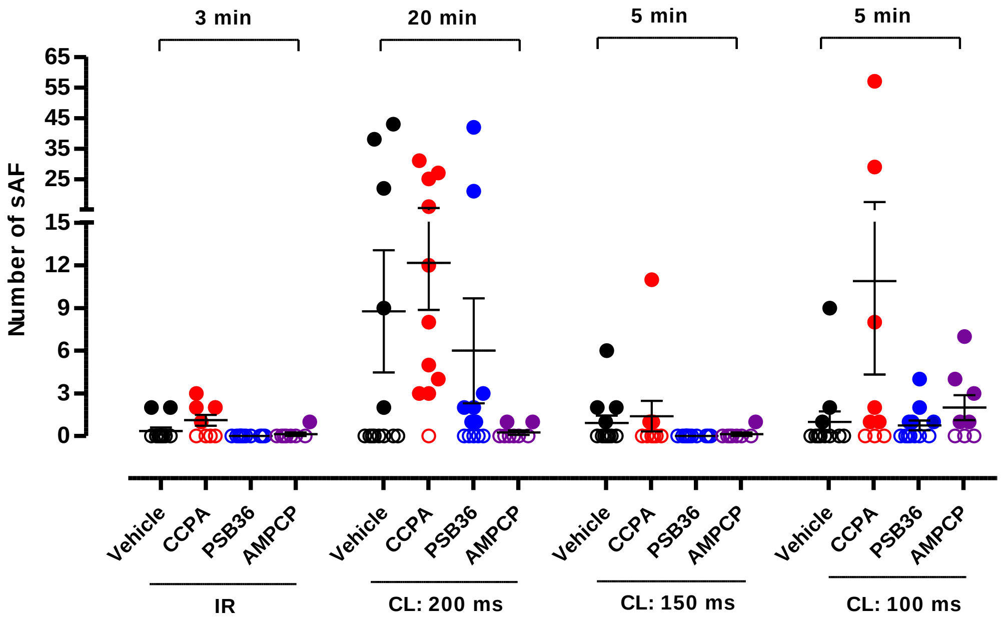

Supplementary Material 7. Number of spontaneous AF episodes occurred during IR and at CL of 200, 150 and 100 ms, respectively. The free run (IR) and the various pacing regimes were maintained according to the time indicated in the graph. At CL of 200 ms, one-way ANOVA test showed a P<0.01 and medians vary significantly (P<0.05).

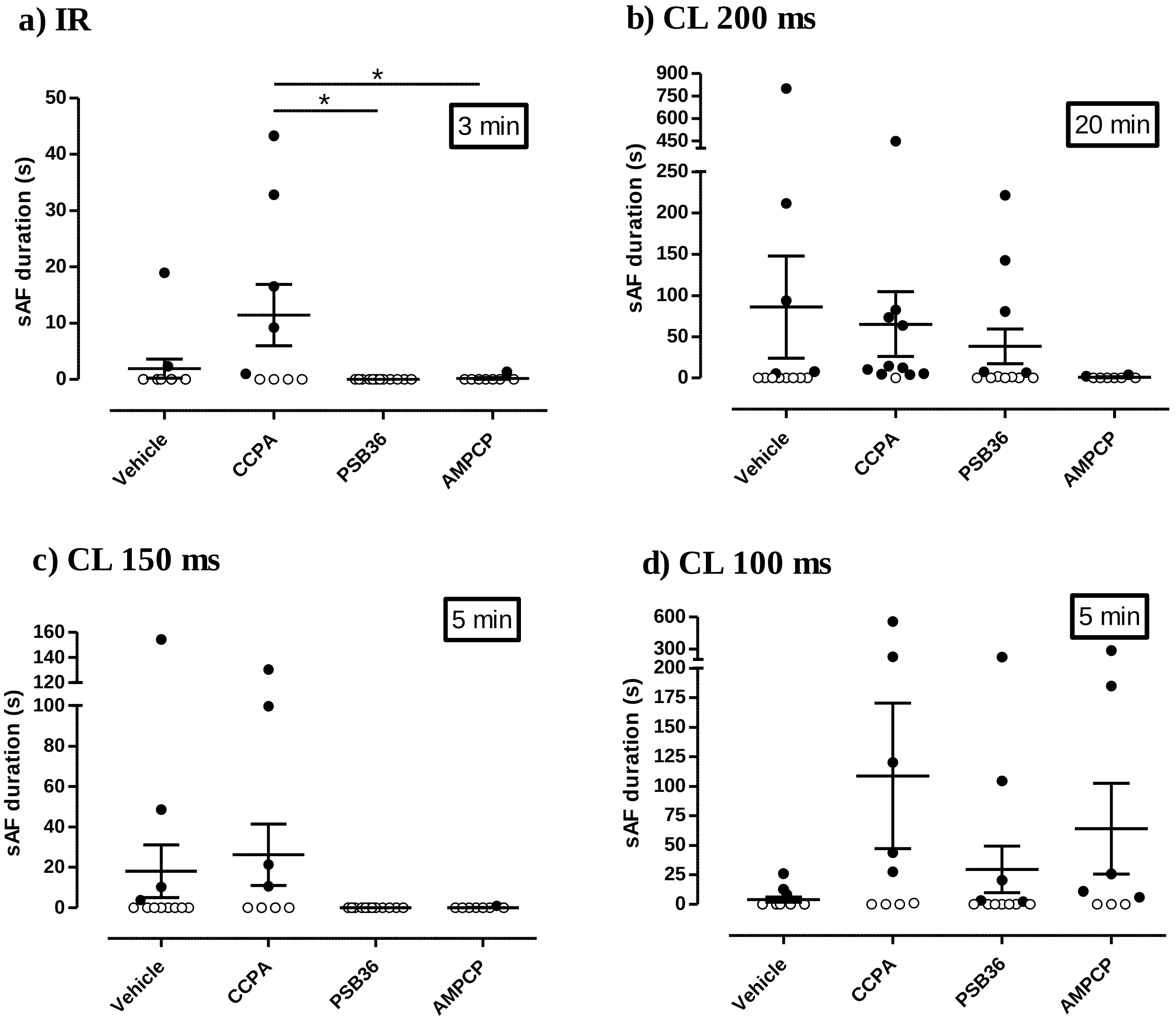

Supplementary Material 8. These graphs represent spontaneous AF (sAF) duration during (**a**) IR period (c. 3 minutes), (**b**) CL 200 ms (c. 20 min), (**c**) CL 150 ms (c. 5 min) and (**d**) CL 100 ms (c. 5 min), respectively. Each dot indicates the summation of all sAF durations at each CL or IR for each experiment. Events that lasted more than 500 ms were taken into consideration as arrhythmic events and are depicted as black dots. White dots represent those experiments in which no sAF event was detected at any time. **a**) One-way ANOVA test showed a $P<0.05$, however there is no significant difference between vehicle and CCPA. In contrast, PSB36 and AMPCP reduce significantly the spontaneous onset of AF when compared to CCPA ($P<0.05$). **b**, **c** and **d** did not show any significant difference among sAF durations.

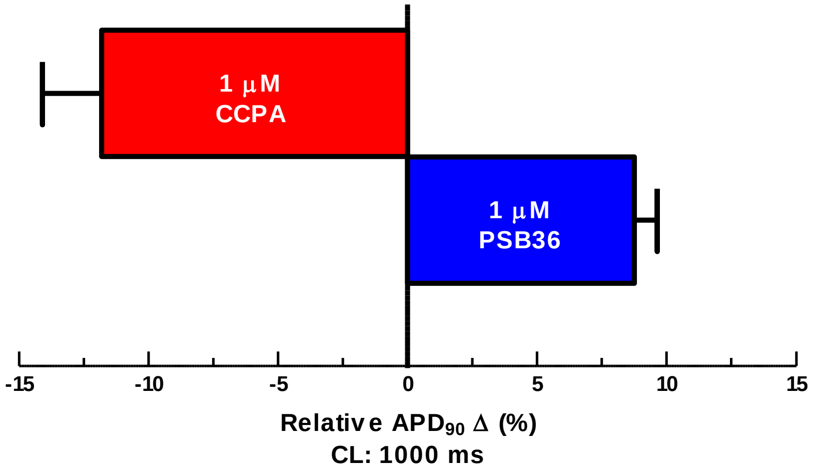

Supplementary Material 9. Relative hRAA APD_{90} variation at CL 1000 ms. CCPA (-11.8 ± 2.3%, n=6) is depicted in red while PSB36 (8.8 ± 0.9%, n=5) in blue.

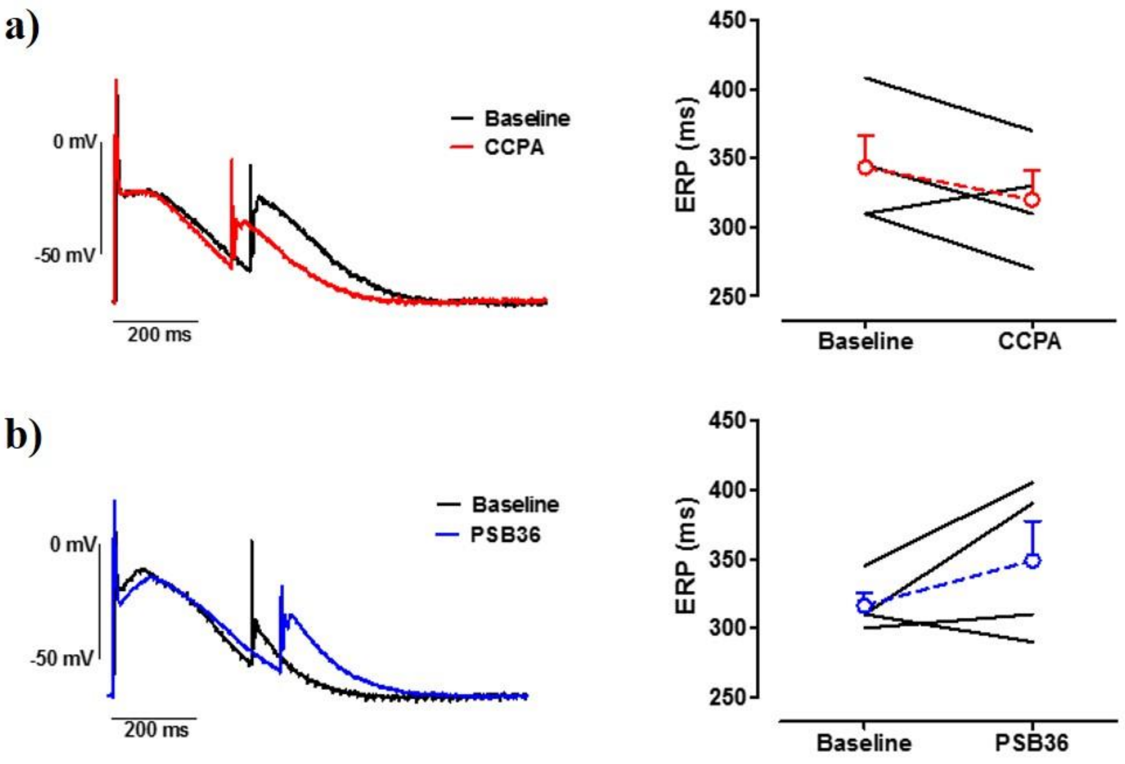

Supplementary Material 10. The effect of A_1-R on ERP in hRAAs. The baseline and the relative treated tissues APs were superimposed. **a)** Baseline 343 ± 23 ms vs. CCPA 320 ± 23 ms; P=0.10, n=4). **b)** Baseline 316 ± 10 ms vs. PSB36 349 ± 29 ms; P=0.13, n=4). RMP in representative panels was normalized.

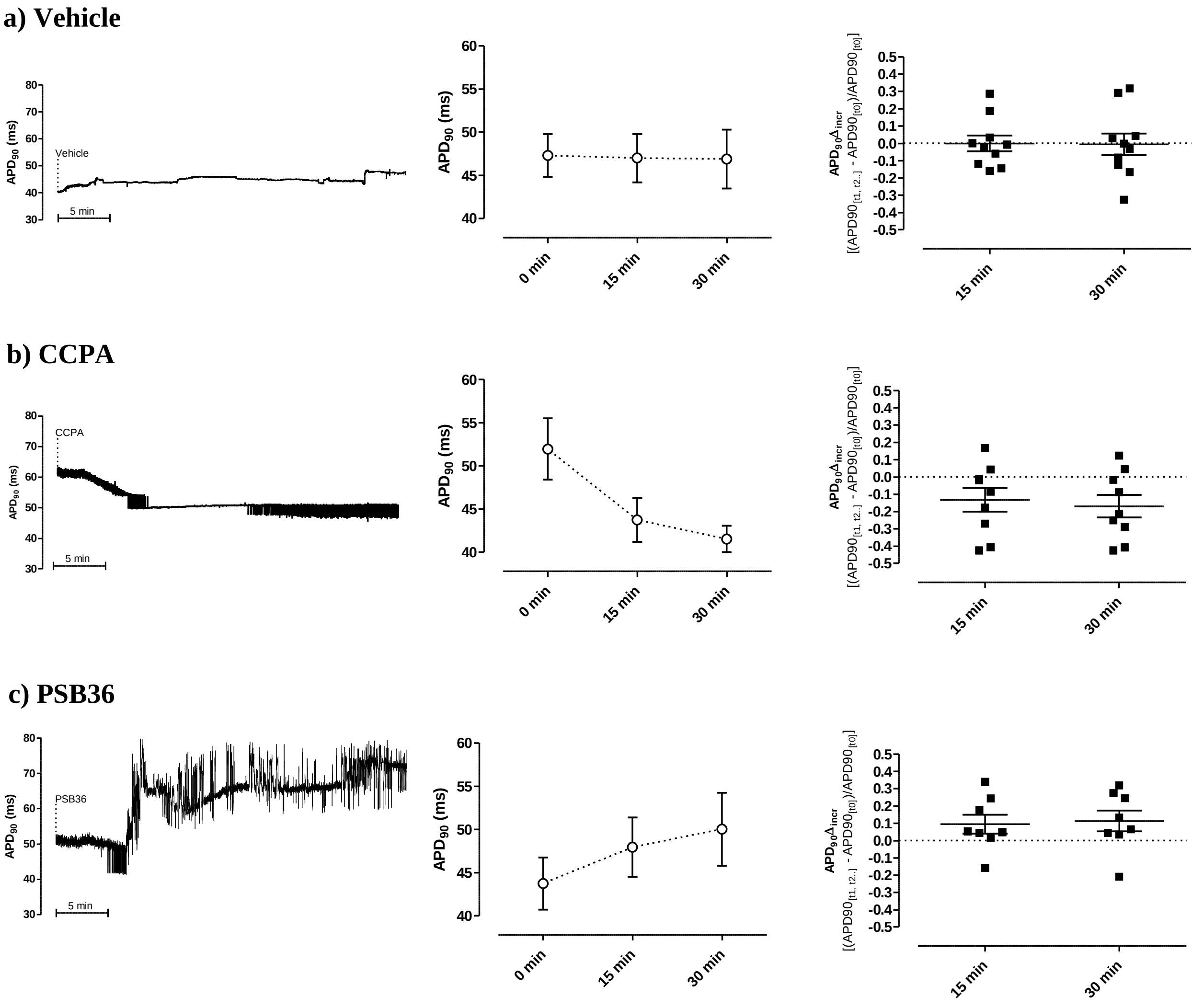

Supplementary Material 11. A_1-R kinetics on the APD_{90}. Graphs show the effect of perfusing the vehicle (**a**), CCPA (50 nM) (**b**) and PSB36 (40 nM) (**c**) on the APD_{90} at a CL of 200 ms. For each group, the first panel indicates a representative perfusion, lasting for 30 min. The second panel shows the APD_{90} measured at 0, 15 and 30 min after drug perfusion. The third panel represents the variation from time zero in APD_{90} at 15 and 30 min. In all cases, variation in APD_{90} between 15 and 30 min is not significant ($p>0.05$). As shown, PSB36 increased DT, which was measured at different time points. In some cases, the applied field current was not sufficient to maintain the pacing regime at a CL of 200 ms. Thus, not all the experiments are represented in **c**.

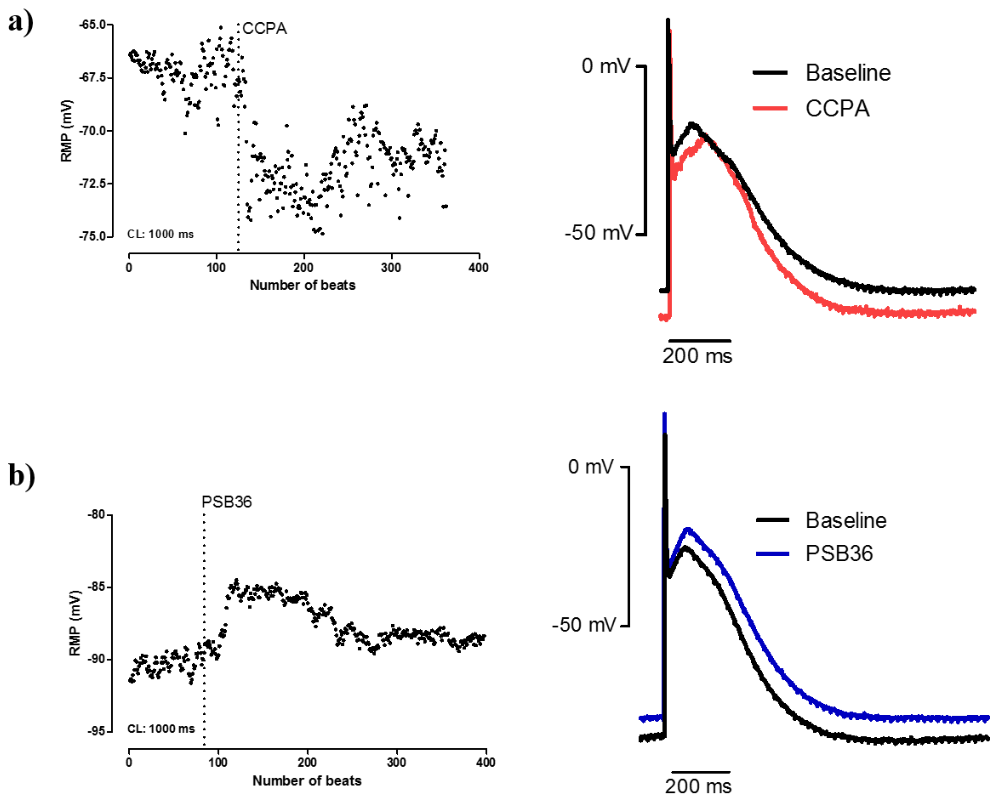

Supplementary Material 12. The effect of A_1-R on RMP in hRAAs. In the first panel, dots represent the RMP at each beat, while in the second the baseline and the relative treated tissues APs were superimposed. **a)** CCPA (1 µM) hyperpolarized the RMP, while **b)** PSB36 (1 µM) induced depolarization of the RMP. In **b** the RMP baseline is considerably hyperpolarized perhaps due to a drift of the system over time.

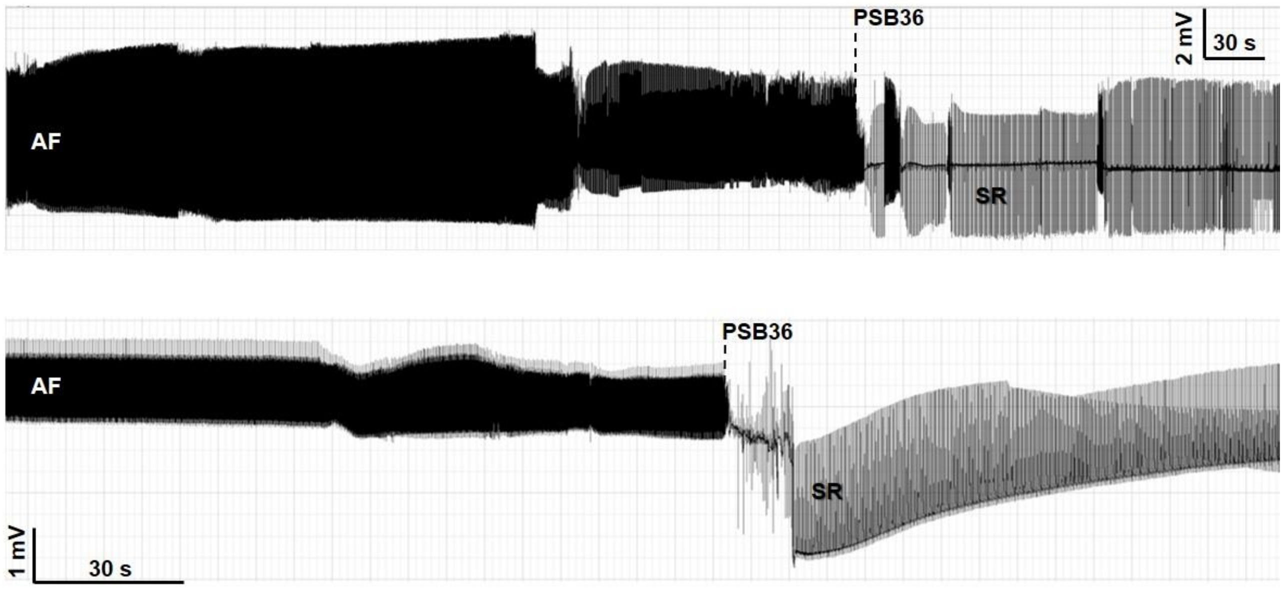

Supplementary Material 13. Atrial MAPs recordings of two pilot experiments on rat isolated perfused hearts. During sustained AF (>5 min), 2 mL bolus of PSB36 (40 ng/mL) was administered via the "*drug injection pathway*" of the Langendorff system. In these conditions, PSB36 fast terminated AF and restored sinus rhythm (SR). Atria were not paced.
